# Novel Air Stimulation MR-Device for Intraoral Quantitative Sensory Cold Testing

**DOI:** 10.3389/fnhum.2016.00335

**Published:** 2016-06-30

**Authors:** Ben Brönnimann, Michael L. Meier, Mei-Yin Hou, Charles Parkinson, Dominik A. Ettlin

**Affiliations:** ^1^Pain Research Lab, Center of Dental Medicine, University of ZurichZurich, Switzerland; ^2^Interdisciplinary Spinal Pain Research ISR, Balgrist University HospitalZurich, Switzerland; ^3^Consumer Healthcare, GlaxoSmithKlineSurrey, UK

**Keywords:** cold air stimulation, QST, fMRI, dentine hypersensitivity

## Abstract

The advent of neuroimaging in dental research provides exciting opportunities for relating excitation of trigeminal neurons to human somatosensory perceptions. Cold air sensitivity is one of the most frequent causes of dental discomfort or pain. Up to date, devices capable of delivering controlled cold air in an MR-environment are unavailable for quantitative sensory testing. This study therefore aimed at constructing and evaluating a novel MR-compatible, computer-controlled cold air stimulation apparatus (CASA) that produces graded air puffs. CASA consisted of a multi-injector air jet delivery system (AJS), a cold exchanger, a cooling agent, and a stimulus application construction. Its feasibility was tested by performing an fMRI stimulation experiment on a single subject experiencing dentine cold sensitivity. The novel device delivered repetitive, stable air stimuli ranging from room temperature (24.5°C ± 2°C) to −35°C, at flow rates between 5 and 17 liters per minute (l/min). These cold air puffs evoked perceptions similar to natural stimuli. Single-subject fMRI-analysis yielded brain activations typically associated with acute pain processing including thalamus, insular and cingulate cortices, somatosensory, cerebellar, and frontal brain regions. Thus, the novel CASA allowed for controlled, repetitive quantitative sensory testing by using air stimuli at graded temperatures (room temperature down to −35°C) while simultaneously recording brain responses. No MR-compatible stimulation device currently exists that is capable of providing non-contact natural-like stimuli at a wide temperature range to tissues in spatially restricted areas such as the mouth. The physical characteristics of this novel device thus holds promise for advancing the field of trigeminal and spinal somatosensory research, namely with respect to comparing therapeutic interventions for dentine hypersensitivity.

## Introduction

Temperature perception and discrimination are part of the body's homeostatic control system that evaluates and integrates internal and external body states. At the cellular level, primary sensory afferents (C- and Aδ-fibers) possess thermoreceptors that transduce distinct temperature stimuli (McKemy, 2007). Innocuous cold perception (cryesthesia; 15 to −30°C) is evoked by distinct nerve fibers that can be categorized as cold thermoreceptors whereas temperature stimuli below 15°C are encoded by cold nociceptors. Peripheral cold temperature receptors belong to the transient receptor potential channel family (e.g., TRPM8 and TRPA1). Furthermore, sodium channels Nav1.8 and transient 4-AP-sensitive K+ currents are also involved in cold related cellular activation and inhibition (McKemy, 2013). Some of these ion channels have also been detected in dental tissue (Story et al., 2003; Patapoutian et al., 2009; Chung et al., 2013). In spite of the diversity of neural substrates for cold signaling, converging evidence suggests that the prime molecular detector of cold is TRPM8, a calcium-permeable cationic ion channel (Madrid and Pertusa, 2013). Both, mammalian dental pulp C- and Aδ-fibers express TRPM8 (Takashima et al., 2007). In molars of transgenic mice, a portion of TRPM8 labeled axons were observed below the odontoblast layers and more interestingly, another subset of TRPM8 fibers crossed the odondoblast layer to extend into dentinal tubules. This observation, taken with functional recordings in animals and psychophysical data in humans suggest that direct stimulation of TRPM8 neurons may play a more important role in dentine cold hypersensitivity than the more popular “hydrodynamic theory.” The latter postulates that dentinal fluid movements evoke neural signaling (Chidchuangchai et al., 2007). By presenting a microscale model of tooth physiology, Lin M. et al. (2014) presented a synthesis of possible thermal transduction mechanisms in teeth from an engineering perspective, highlighting the activation of stress-sensitive ion channels on nociceptors by cooling effects.

On the brainstem level, unmyelinated, and small myelinated TRPM8 neurons from intra- and perioral areas predominantly project onto second order neurons in the rostral trigeminal sensory nuclei (Kim et al., 2014). Likely due to lack of cold stimulation techniques applicable in a magnetic resonance scanner environment, it remains an open question which areas of the human brain are involved in the processing of dentine cold hypersensitivity (Meier et al., 2012).

In the clinical environment, investigations on dentine hypersensitivity (DH) rely on a variety of qualitative or semi-quantitative dental stimulation techniques. These methods encompass Yeaple® pressure probes (Chabanski et al., 1997), percussion testing, bite stress tests, water syringe, and piezoelectric magnetomechanical devices for applying vibrotactile stimuli to teeth. Other approaches include air application from triple air syringes or cold sprays at freezing temperatures. Cool or warm spatula have also been used for sensory testing. Non-contact air puff stimulators comprising pneumatically and mechanically driven devices are mainly used in research settings (Puce et al., 1995; Wallois et al., 1997; Keller et al., 2002; Briggs et al., 2004; Ettlin et al., 2004; Moana-Filho et al., 2010; Pigg et al., 2010; Lindstedt et al., 2011; Svensson et al., 2011; Ahn et al., 2012; Brügger et al., 2012; Meier et al., 2012).

Current experimental thermal stimulation tools are limited in their ability to produce computer-controlled, graded, non-contact and cold temperature stimuli for quantitative sensory testing (QST) and none is applicable in a high magnetic field environment such as an MR-scanner. For extraoral air stimulation Servos et al. (1998) used an MR-compatible air stimulation device capable of applying very short air puffs on the skin at room temperature. Using the same temperature range, Meier et al. (2012) used an MR-compatible air stimulation device for investigating DH subjects' brain responses.

Arguably, cold temperature stimuli most closely imitate naturally occurring DH. Therefore, the aim of this study was (1) to design and construct a novel MR-compatible and computer-controlled dental stimulation device capable to operate in a broad range of cold temperatures, and (2) to show its MR-feasibility in a single DH subject.

## Materials and methods

The experiment was previously approved by the local ethics committee (KEK-ZH-Nr. 2010-0347) and the volunteer signed an informed consent.

### System design

**Figure 1:**
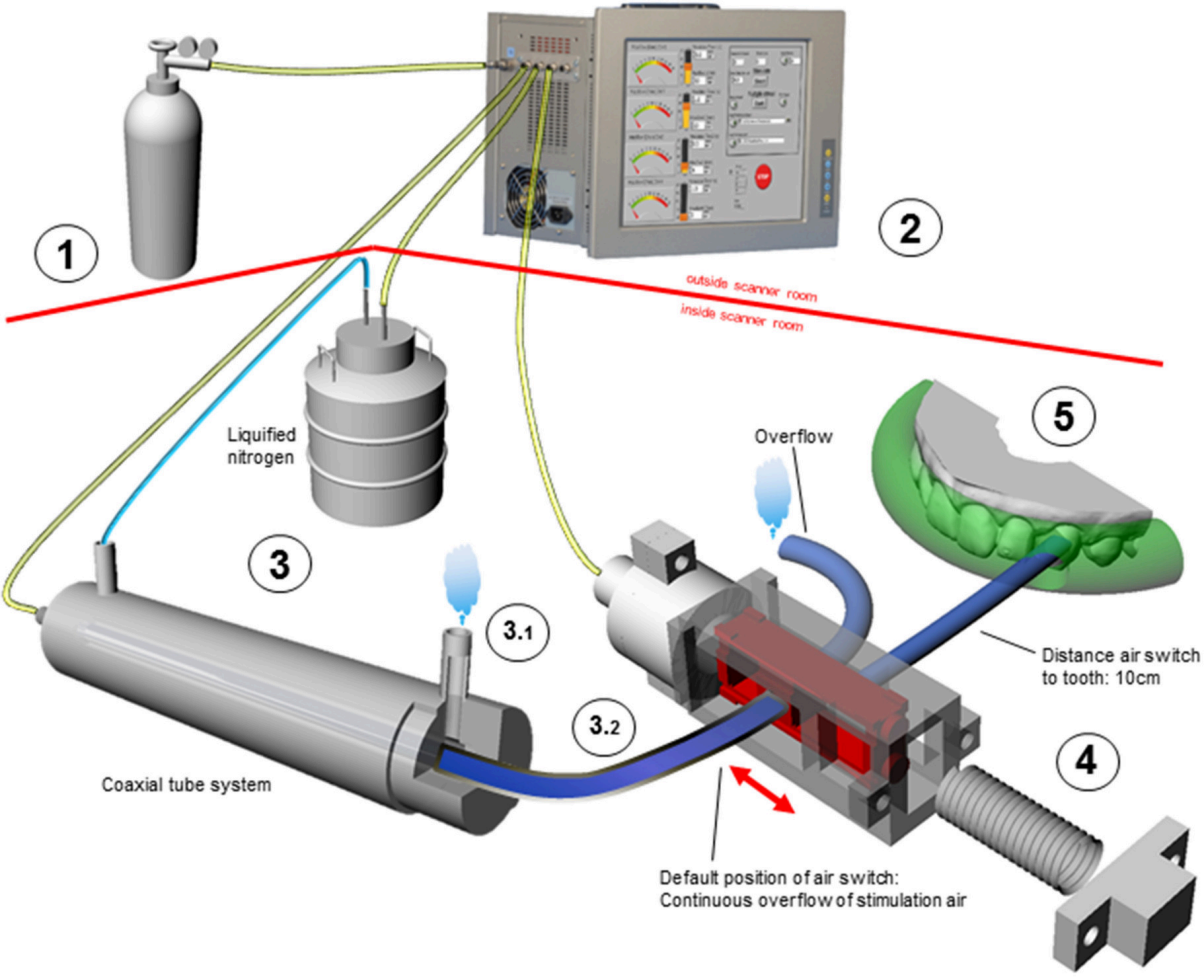
**Schematic display of the CASA components (graphical objects not shown in proportional scale)**. Outside scanner room: (1) Air source (2) Computer-controlled air jet delivery system with three outlets: cooling system, stimulation air, and pneumatic control of air switch. Inside scanner room: (3) Cold exchanger [note: the two gas circuits for cooling (3.1) and stimulation (3.2) are completely separated] (4) Air switch composed of mobile slider (in red) and spring (5) Only one target tooth is exposed to air while the others are shielded by impression material (transparent green). The various colors of the connecting tubes reflect different air temperatures (T°) in the tubes: room T° (yellow); approx. −196°C (light blue); target T° for tooth stimulation (dark blue).

The cold air stimulation apparatus (CASA) was designed to include four tube-connected components (Figure 1): (1) An air source that does not condense at very low temperatures and that feeds into a computer-controlled air jet delivery system, (2) a cold exchange system (CE), and (3) an air switch that directs the cold stimulus to and away from the target tooth. A temperature sensor served for monitoring stimulation temperature.

Instead of regular air, compressed nitrogen of high purity was used in order to prevent tube condensation and freezing upon gas cooling.

#### Air jet delivery system

A modified version of the previously developed multi-injector AJS was used that consists of four computer-controlled channels of which three were used for the CASA (Megias-Alguacil et al., 2008). This system is able to apply air flow rates between 0.3 and 20 l/min in steps of 0.1 l/min. One channel delivered nitrogen steam from a cryotank to the CE (see Section Cold Exchange System and Temperature Regulation, Figure 1). A second channel served to deliver temperature-graded air puffs to the target tooth. A third channel controlled the position of the air switch delivering the stimulus to the target tooth (see Section Air Switch and Dental Splint).

#### Cold exchange system and temperature regulation

The stimulation air was cooled by the CE which consisted of a 1.75 m long coaxial tube system made of non-ferromagnetic pure stainless steel (Inox-steel-technology, Edelstahl-Anlagebau, CH-3645 Thun, Switzerland). Its outer chamber had two outlets for circulatory flow of nitrogen steam (−196°C) delivered by a liquid nitrogen tank (Figure 1). The temperature of the stimulation air passing through the inner chamber was controlled by adapting the flow rate of the nitrogen steam in the outer chamber by the AJS. To avoid subject contact, the tube releasing the outflowing nitrogen steam was positioned away from the subject. All tube connections were made of cryoresistant Polytetrafluorethylen (PTFE) and had an inner diameter of 4 mm (Maagtechnic AG, CH-8600 Duebendorf, Switzerland). MR-compatible pure stainless steel connections served as tube connectors (Swagelock®, Arbor AG, CH-5443 Niederrohrdorf, Switzerland). Highly flexible elastomeric material was used for maximum thermal tube insulation (Armaflex®, Regisol AG, CH-3292 Busswil, Switzerland). For monitoring stimulation air temperature and humidity, a fiberoptic temperature sensor (Reflex® 4 channel, Neoptix®, Canada, Switzerland) and a humidity sensor (SHT21, Sensirion AG, CH-8712 Staefa, Switzerland) were positioned after the air switch.

#### Air switch and dental splint

Due to the air's low thermal capacity, turning off the air flow would result in its immediate and uncontrolled warming. To avoid this problem, the air flow was kept constant at a given flow rate and a custom made MR-compatible air switch regulated its direction to and away from the tooth (Figure 1). This air switch was designed by means of 3D design software (Rhino 5.0®) and subsequently printed on a 3D printer using Rigid Opaque Gray Material (Objet Eden 260 V®, Stratasys, Eden Prairie, MN, 55344 USA). The time lag of the slider position change to the target tissue was estimated using the formula:

$$\Delta t=\frac{V}{{}^{\wedge }V}=\frac{A^{*}L}{{}^{\wedge }V}$$

where Δt is the lag from switch operation to tooth stimulation, V the stimulation air tube's volume (length 0.1 m, diameter 2 mm), ^∧^V the volume flow (13 l/min.), A the tube's cross section area and L the tube's length (0.1 m).

The air switch was attached to the MR head coil by plastic cable ties. In non-stimulation mode, the slider was held in position by a custom-designed non-ferromagnetic phosphor-bronze compression spring (Favre-Steudler SA, CH-2504 Biel-Bienne, Switzerland). For tooth stimulation, the spring force was counteracted by air pressure so that the cold air was directed toward the tooth (Figure 1).

To secure stimulation of one single target tooth, all other teeth were tightly covered by a subject tailored dental splint made of thixotropic vinyl polysiloxane (Blue mousse®, Parkell, Inc., Edgewood, NY 11717 USA).

#### Monitoring of target tooth temperature

A supplemental *ex vivo* cold air stimulation experiment was performed to assess cooling effects on the target tooth. For this purpose a temperature flow sensor was placed on the palatal surface while directing cold air of −35°C to the labial surface for 10 s (gSKIN®, GreenTEG, CH-8005 Zürich, Switzerland).

### MR feasibility testing

**Figure 2:**
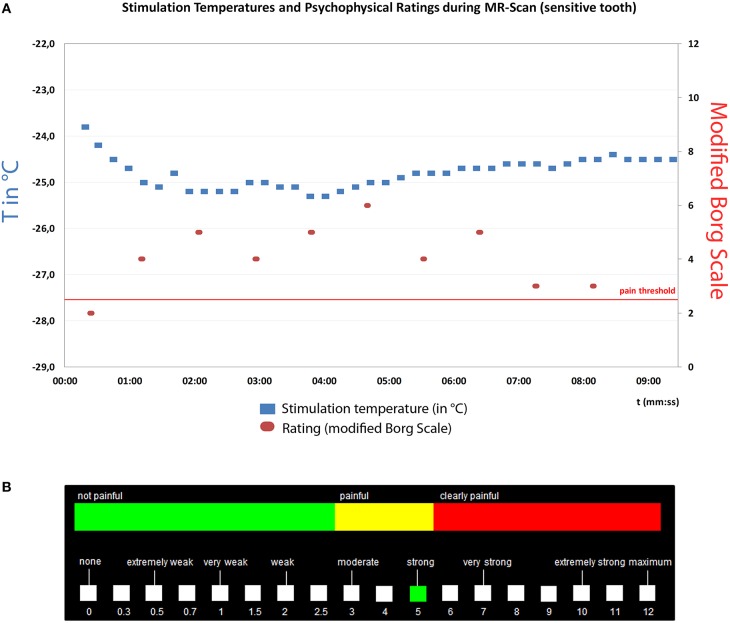
**(A)** Psychophysical testing of a sensitive tooth during MR scan in a single subject. Left x-axis: Stimulation temperature in °C (blue dots), Right y-axis: Subject ratings on modified BORG scale (red dots), x-axis: time (minutes). Pain threshold was defined as BORG score >2.5. **(B)** The BORG scale was presented on a screen and consisted of non-linearly distributed numerical (from 0 to 12), verbal (from none to maximum) and color-coded (green, non-painful; yellow, painful; red, clearly painful) descriptors of the sensory perception.

To test the functioning of all system components and to explore supraspinal effects of the cold stimulus, an fMRI experiment was performed on a single DH subject (female, 25 years). For tooth screening, the sensitivity of the front teeth and premolars was qualitatively assessed by directing air to the teeth at room temperature (24.5°C ± 2°C) at a flow rate of 10 l/min. The most sensitive tooth was determined both by using an oral pain report and the Schiff scale for dentine hypersensitivity (Schiff et al., 2006). Next, QST was performed by using the CASA. The air temperature applied to the target tooth was adjusted in 2°C increments (±1°C) in order to identify its pain threshold by the ascending methods of limits (stimulus duration of 3 s). Every stimulus was rated on a modified version of the Borg Scale (Borg, 1998). This scale consisted of non-linearly distributed numerical (from 0 to 12), verbal (from none to maximum) and color-coded (green = non-painful, yellow = painful, red = clearly painful) descriptors of the sensory perception (Figure 2B). We aimed at identifying the temperature that evoked a clear but tolerable pain rated 6 on the modified BORG scale. The subject's perception of the stimulus quality was assessed by both, a free subject report and by forced choice responses for descriptors presented by Beissner (Beissner et al., 2010).

During MR-scanning the subject was then exposed to 40 cold air stimuli (duration 3 s) at the predetermined target temperature applied to the sensitive tooth with an interstimulus interval of 10 s (Figure 2A). To monitor stable stimulus perception during the fMRI measurement, the subject was asked to rate every fourth stimulus on the modified Borg Scale presented on a screen (Figure 2B) by operating a trackball (fORP 932 response package, Cambridge research systems, Rochester, Kent, ME2 4BH, UK). During non-rated stimulation a green cross (4 × 4 cm) was presented in the middle of a black screen.

#### Scanning parameters

All measurements were performed on a 3-T whole-body MRI system (Philips Ingenia, Best, the Netherlands). For functional scanning, a blood oxygen level dependent (BOLD) sensitive single-shot gradient echo planar imaging sequence was used to acquire 33 axial slices, covering the entire cerebrum and cerebellum, using a 32- channel head coil (dStream Head 32ch coil, Philips). Parameters: echo time 30 ms, flip angle = 75°, repetition time 2586 ms, slice thickness 4 mm, inter-slice gap = 0 mm, field of view 230 mm and matrix size in plane 128 × 128, resulting in a voxel size of 2.75 × 2.75 × 4 mm. Six dummy scans were first acquired and discarded to reach steady state magnetization.

In addition, 180 high-resolution T1 weighted axial slices (spoiled gradient echo) were acquired with TR = 20 ms, flip angle = 20°, voxel size = 0.98 × 0.98 × 1.02 mm, FOV = 22 cm, matrix = 224 × 187.

#### Image preprocessing and event related analysis

SPM12 (version 6470) running on MatLab 2015a (The MathWorks, Massachusetts, USA) was used for the brain activity analysis (http://www.fil.ion.ucl.ac.uk/spm/software/spm12/). Functional EPI volumes of each subject were corrected for differences in head motion, spatially normalized according to the Montreal Neurological Institute (MNI) space and finally smoothed with a 8 mm full-width at half-maximum (FWHM) Gaussian kernel. To control for confounding head movement effects, individual movement parameters (translations in x, y, and z-direction, as well as rotations around x, y, and z axis) were implemented in the 1st level model as regressors of no interest. Excessive head motion was defined as a dislocation of more than once the in-plane voxel resolution (>2.75 mm). For removing the low frequency noise, a high-pass filter with a cut-off of 128 s was used. The Borg Scale rating trials were modeled as regressors of no interest to exclude brain activation related to motor activity, resulting in 30 trials of interest (noxious cold air stimulation). These trials were modeled as boxcar regressors and convolved with the standard canonical hemodynamic response function (HRF) as implemented in SPM12. For the 1st level analysis, the general linear model (GLM) was fitted by a design matrix composed of the onsets and duration (3 s) of the noxious air stimuli.

Activations were considered as statistically significant when falling below a statistical threshold of *p* <0.05, corrected for multiple comparisons using voxel-wise family-wise error correction (FWE). Finally, a T-contrast was computed to investigate whole-brain activity based on the contrast “noxious cold air stimulation vs. baseline (no stimulation).” Peak coordinates of the clusters were extracted and the respective anatomical locations were identified by means of the Automated Anatomical Labeling (AAL) atlas using the WFU pickatlas toolbox (http://fmri.wfubmc.edu/software/pickatlas).

## Results

### Stimulus characteristics

Within the MR scanner, CASA allowed the intraoral application of air stimuli at temperatures ranging from room temperature to <−40°C at flow rates ranging from five to a maximum of 17 l/min. Minimal stimulation duration was 0.6 s, with a maximum of several minutes. The CE system allowed a manually controlled temperature adjustment within several minutes. Once the stimulus air temperature was reached, it could be kept constant with a tolerance of ±2°C for a maximum of 9 min (Figure 2).

### Air switch and stimulation tooth

The air switch could redirect a constant flow of cold air toward a target tooth at temperatures reaching <−40°C for a short time range (several seconds). For longer lasting stimulation periods a minimum of −35°C turned out to be appropriate to avoid freezing of the air switch. Flow rate measurements revealed an overall reduction of 0.3 l/min (SD 0.24 l/min) of the preset Air Jet output flow rate compared to the flow rate reaching the target tooth due to minor air loss within the air switch. The approximate air travel time from the switch valve to the target tooth lasted 222 ms. This value was calculated by adding up the 220 ms switch sliding time (mean of five measurements using a 10 m long non-curled silicone tube of 4 mm diameter and the air flow set to 13 l/min) with the 2 ms air column movement time (see formula in Section Air Switch and Dental Splint).

### Temperature flow measurement on tooth

By placing a temperature flow sensor (gSKIN®, GreenTEG, CH-8005 Zürich) on the palatinal surface of an extracted tooth, temperature flow measurements during cold air stimulation applied to the labial surface revealed that the temperature gradient between the opposing tooth surfaces remained constant across a 10 s stimulation period at −25°C.

### Behavioral results and MR feasibility testing

#### Behavioral results of DH subject

Repeated psychophysical assessment of the sensitive tooth (tooth 12) revealed a target temperature of −25°C at a flow rate of 16 l/min with a stimulus duration of 3 s for achieving a moderately painful sensation. The stimulus quality was described by subjects as “stinging” and “icy” like “cold air in wintertime.” The perception was only felt in the target teeth and not in adjacent tissues. No tactile perception was reported by subjects. During scanning the tooth 12 was exposed to 40 stimuli at a temperature of −24.8 ± 0.3°C (Figure 2A). The mean rating of sensitive tooth stimulations on the modified Borg scale was 4.1 (SD 1.2, Figure 2A).

#### fMRI results

**Figure 3:**
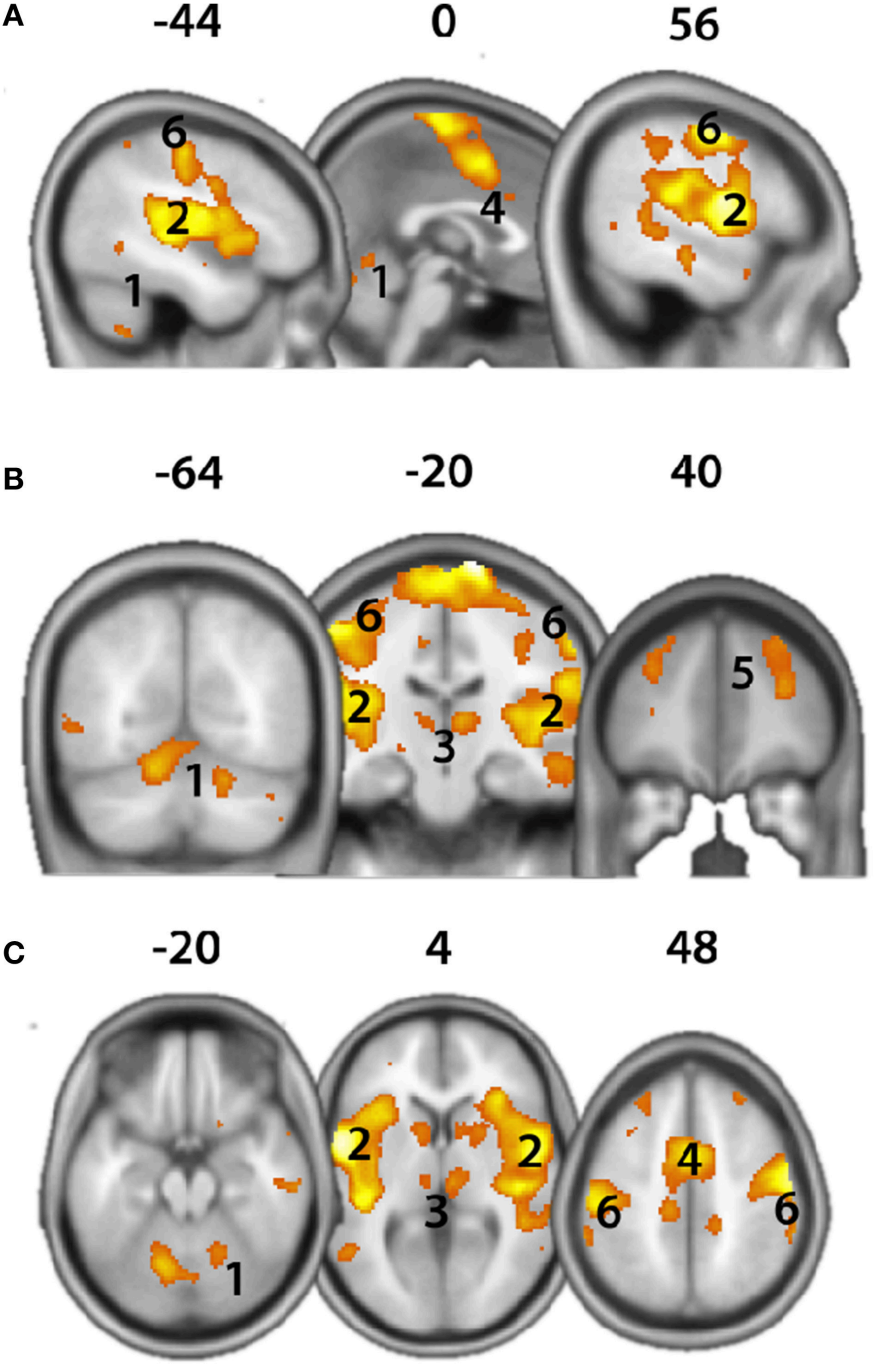
**Brain activity evoked by noxious cold air stimulation (T-Contrast “Noxious cold air stimulation >no stimulation,” ***p*** < 0.05, FWE-corrected) evoked neural activity in wide regions of the brain**. **(A)** Sagittal view; **(B)** coronal view; **(C)** axial view. 1, Cerebellum; 2, Insula; 3, Thalamus; 4, cingulate cortex; 5, Frontal cortex; 6, somatosensory cortex. X,Y,Z-coordinates are shown in MNI space.

Head motion range was minimal (<1.8 mm). The brain activity analysis of noxious cold air stimulation revealed neural activity in an extensive network of brain regions. Significant brain activity could be observed in the cerebellum, in anterior-to-posterior portions of the insular cortex, in the primary and secondary somatosensory cortex, in the thalamus, in cingulate cortices and in frontal regions (Figure 3, *p* < 0.05, FWE-corrected).

## Discussion

Since transduction mechanisms are determined by stimulus characteristics, QST of a specific stimulus type is an ideal method to assess sensory function and dysfunction. The main finding of this study demonstrated that the CASA is a useful tool for QST of sensitive teeth. It enables application of computer-controlled, intraoral and natural non-contact stimuli in the range of room temperature to −35°C in a 3 Tesla MR-environment. Further, it allowed recording of BOLD signals in response to painful intraoral cold air stimuli.

Principally, our fMRI paradigm can be modified for many research questions related to cold stimulus induced brain responses. In order to show the fMRI feasibility of our novel apparatus we chose a well-established fMRI paradigm based on model dependent and task-related brain activity. Using more sophisticated methods in future experiments such as model-free and/or time course analysis (Cauda et al., 2014) in combination with our novel apparatus will likely advance the understanding of trigeminal cold pain sensation. The current task related analysis of fMRI responses yielded brain activation typically associated with the “neuromatrix,” a brain network that is involved in the processing of salient stimuli such as pain (Iannetti and Mouraux, 2010). Specifically, we observed significant neural responses in the thalamus, primary and secondary somatosensory cortices (Data Sheet 1), insular and cingulate cortices, frontal cortices and the cerebellum (Peyron et al., 2000; Apkarian et al., 2005; Iannetti and Mouraux, 2010; Moulton et al., 2010; Duerden and Albanese, 2013). Furthermore, current initial results show agreement with other reports focusing on brain activity of dental pain (Lin C. et al., 2014). Thus, the feasibility and MR-compatibility of the CASA have been confirmed.

Neuroscience aims at understanding nerve function along the entire neuraxis from stimulus transduction to central perception. Previous available experimental thermal stimulation tools were limited in their ability to apply reproducible computer-controlled, graded non-contact and cold temperature stimuli. None was applicable in a high magnetic field environment such as an MR-scanner. Natural cold air stimuli may best mimic clinically relevant pain experienced by DH patients. Furthermore, simultaneous recording of brain activity by means of fMRI might broaden our knowledge of DH-related central processes. A first step in this direction was done by Meier et al. (2012) who stimulated sensitive teeth with air stimuli at room temperature within an MR environment. By comparison, our novel CASA opens up new possibilities by offering a broader stimulus temperature range up to freezing temperatures (<−35°C) thus providing more appropriate stimuli for investigating tooth sensitivity such as DH. Considering the complexity of peripheral signal transduction mechanisms, the application of graded non-contact cold stimuli might be helpful to further elucidate mechanisms of dental signal transduction (Lin M. et al., 2014). Finally, CASA can also be applied to other body parts and therefore allows novel opportunities for investigating supraspinal mechanisms of painless and painful cold perceptions.

### Limitations

A limitation of the CASA is its time requirement for manual temperature calibration to reach the target temperature. It is shortened by progressively advanced operator experience. Future studies will have to demonstrate if the single-subject fMRI data observed in this report can be extrapolated to larger population groups.

## Author contributions

BB: Development MR-compatible cold air stimulation device, recruitment, operation cold air stimulator during MR-measurement, statistical analysis. MM: MR-measurement, statistical analysis. MH: dental examination of subject. CP: Study design. DE: PI, Study design, development MR-compatible cold air stimulation device.

### Conflict of interest statement

This research project was supported by GlaxoSmithKline, Consumer Healthcare, Weybridge, UK. CP is an employee of the sponsor, GSK. All other authors declare that the research was conducted in the absence of any commercial or financial relationships that could be construed as a potential conflict of interest.
