# Supplementary material for: Novel Air Stimulation MR-Device for Intraoral Quantitative Sensory Cold Testing
Source: Front Hum Neurosci. 2016 Jun 30;10:335. doi: 10.3389/fnhum.2016.00335 (PMC4928459; doi:10.3389/fnhum.2016.00335)
Supplement: Supplementary file 1 [file DataSheet1.pdf]

Supplementary material for manuscript

Novel air stimulation MR-device for intraoral quantitative sensory cold testing

Ben Brönnimann[1*], Michael L Meier[1,2], Mei-Yin Hou[1], Charles Parkinson[3], Dominik A Ettlin[1]

Setting of sensors for stimulus temperature evaluation at target tooth

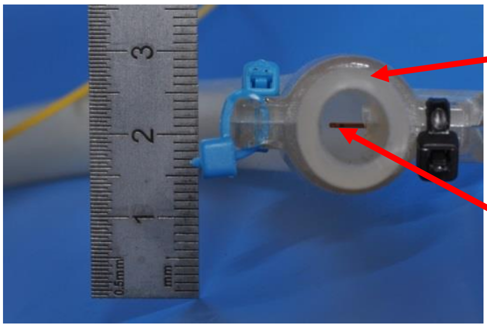

Overflow tube (cross sectional view)

Fig. I: Position of fiberoptical 1 sensor in **overflow tube** monitoring the air flow in the no stimulation position of the slider.

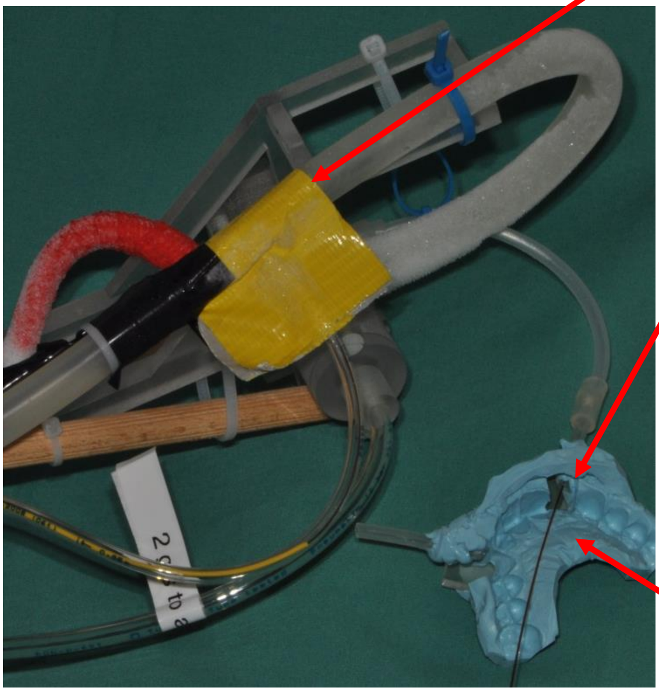

Fig. II: Position of fiberoptical sensor 2 at the **target tooth's exit of stimulation air tube** (here: tooth 12, tube diameter 2 mm). Due to restricted spatial condition at target tooth site, this sensor was only mounted for the evaluation of the stimulation air temperature, but not during the subject's tooth stimulation.

Custom made splint

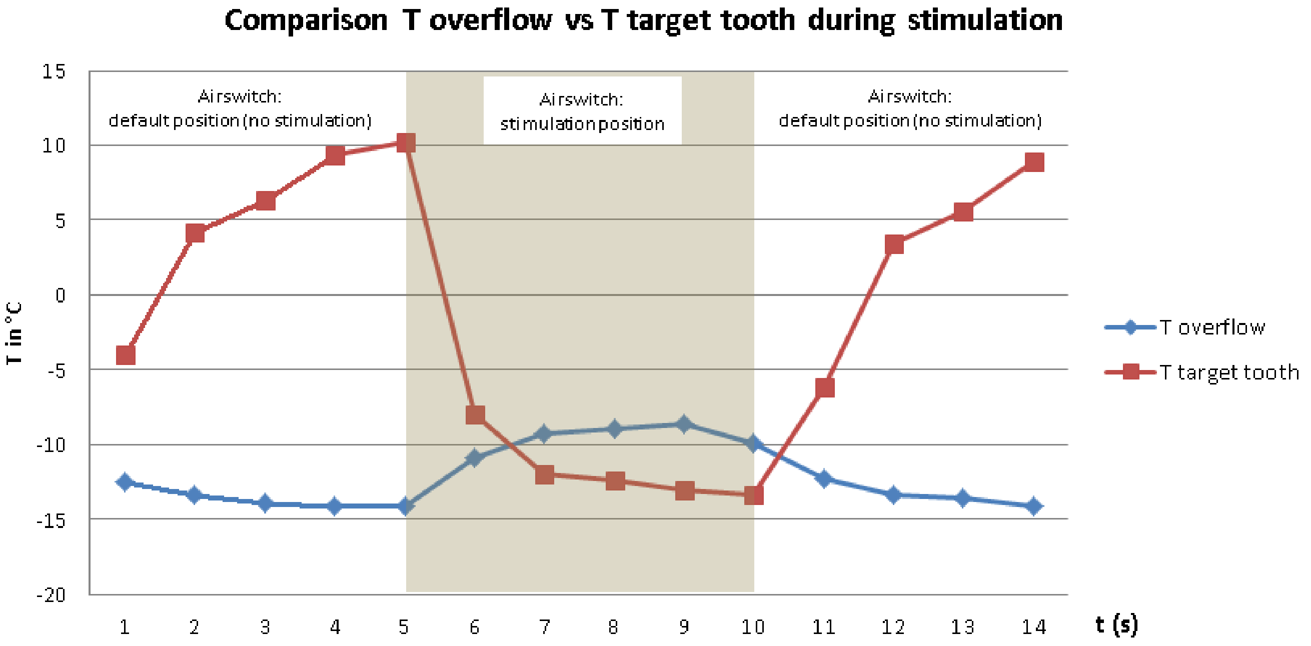

Graph I: Temperature course of a 5 second stimulus phase (shaded area). Red dotted line shows temperature course of air at target tooth's site (sensor 2). Blue dotted line depicts temperature course in overflow tube. Max. time resolution of temperature monitoring: 1 s.
